# LOX1-mediated supramolecular self-assembly nanomedicine for microsatellite-stable colorectal cancer towards reactivating anti-tumor immunity

**DOI:** 10.3389/fimmu.2025.1737031

**Published:** 2025-12-18

**Authors:** Xin Wei, Yuyan Guo, Jianyuan Lei, Na Chen

**Affiliations:** 1 Department of Medical Oncology, The First Affiliated Hospital of Xi’an Jiaotong University, Xi’an, China; 2 Department of Radiation Oncology, The Second Affiliated Hospital of Xi’an Jiaotong University, Xi’an, China; 3 Department of Pathology, Shaanxi Provincial People’s Hospital, Xi’an, China; 4 Department of Rehabilitation Medicine, The First Afffliated Hospital of Xi’an Jiaotong University, Xi’an, China

**Keywords:** colorectal cancer, LOX1, macropinocytosis, PD-1, tumor microenvironment, β-catenin

## Abstract

**Introduction:**

Microsatellite stable colorectal cancer (MSS-CRC) is referred to as an immune desert-type tumor. Emerging studies have demonstrated that inhibition of the Wnt/β-catenin pathway can enhance the anti-tumor immune response. This research focuses on synthesizing the human serum albumin (HSA) -based Wnt inhibitor GHSACA and assessing its internalization pathway and therapeutic effectiveness in conjunction with PD-1 antibody for treating MSS-CRC.

**Methods:**

Glucosamine was covalently conjugated to HSA via EDC/NHS activation to generate GHSA, which was subsequently coupled with carnosic acid (CA) -Wnt pathway inhibitor, to synthesize GHSACA. For *in vitro* studies, LOX1- and CD44- deficient MC38 and CT26 colon cancer cell lines, validated by RT-qPCR, were used to investigate the cellular uptake mechanism and macropinocytic activity of GHSACA using flow cytometry. The effectiveness of GHSACA in targeting tumors *in vivo* was assessed in CT26 tumor-bearing BALB/c mice via fluorescence imaging. Therapeutic efficacy was assessed in MSS-CRC patient-derived xenograft (PDX).

**Results:**

RT-qPCR confirmed efficient knockdown of LOX1 and CD44 in both MC38 and CT26 cell lines. Cellular uptake assay demonstrated that GHSACA internalization is predominantly mediated by the LOX1-dependent macropinocytosis pathway. *In vivo* fluorescence imaging revealed sustained accumulation of GHSACA in tumor and rapid clearance from normal organs. In the PDX model, GHSACA monotherapy significantly suppressed tumor growth (TGI = 59.3%). The combination with PD-1 antibody (G&P group) resulted in further enhancement of antitumor efficacy (TGI = 87.9%). TUNEL assays showed the most pronounced induction of tumor cell apoptosis in the G&P group. Immunohistochemical analysis demonstrated that GHSACA suppressed the Wnt/β-catenin signaling cascades, alongside with lower Ki67 expression. The G&P combination increased PD-L1 expression and significantly boosted Granzyme B-positive cytotoxic immune responses. Immunofluorescence double staining showed the highest infiltration of CD3^+^/CD8^+^ cytotoxic T lymphocytes of the G&P group, with a concurrent decrease in CD4^+^/CD25^+^ regulatory T cells. GHSACA was found to have favorable systemic biocompatibility and safety.

**Discussion:**

GHSACA achieves efficient targeted cellular internalization via a macropinocytosis pathway regulated by the LOX1 receptor rather than the CD44 receptor, simultaneously inhibiting the Wnt/β-catenin signaling while activating anti-tumor immune responses. It provides a highly promising translational therapeutic approach for overcoming immune resistance in MSS-CRC.

## 1 Introduction

Colorectal cancer ranks among the malignancies with high incidence and mortality rates globally (1). Among these, microsatellite-stable colorectal cancer (MSS-CRC) constitutes the vast majority of cases. Due to its “immune desert” tumor microenvironment, MSS-CRC shows limited responsiveness to immunotherapy with PD-1 inhibitors. This has emerged as a significant challenge in clinical treatment (2, 3). There is an urgent need for new strategies to remodel the tumor immune microenvironment and increase MSS-CRC’s sensitivity to immunotherapy.

The abnormal activation of the Wnt/β-catenin signaling pathway is a key factor in the development and progression of colorectal cancer. Persistent activation of this pathway directly promotes tumor cell proliferation, inhibits differentiation, and impedes dendritic cell recruitment and activation by suppressing chemokine secretion like CCL4. Consequently, this leads to a notable deficiency in the infiltration of cytotoxic T lymphocytes at the tumor site, thereby establishing an immunosuppressive microenvironment (4, 5). Targeting the Wnt/β-catenin pathway is a promising therapeutic strategy that can directly inhibit tumor growth and indirectly enhance the anti-tumor immune response (6). However, drugs aimed at the Wnt/β-catenin pathway have consistently encountered challenges in clinical development. Among these challenges, the dearth of an efficient tumor-targeted delivery system is a primary contributor to their substantial systemic toxicity and narrow therapeutic window (7). Nanomedicines present a viable approach to address this conundrum. Through meticulous carrier design, nanomedicines can effectuate the selective enrichment of drugs at tumor sites, augmenting therapeutic efficacy while minimizing adverse effects (8). In previous research, we developed a novel nanomedicine called GHSACA, which is based on glycosylated human serum albumin (9). This nanomedicine employs glycosylated human serum albumin (GHSA) as a carrier to conjugate glucosamine with carnosic acid (CA), a small molecule inhibitor of the Wnt/β-catenin pathway. The aim is to exploit the increased nutrient uptake of tumor cells for targeted drug delivery.

**Figure 1:**
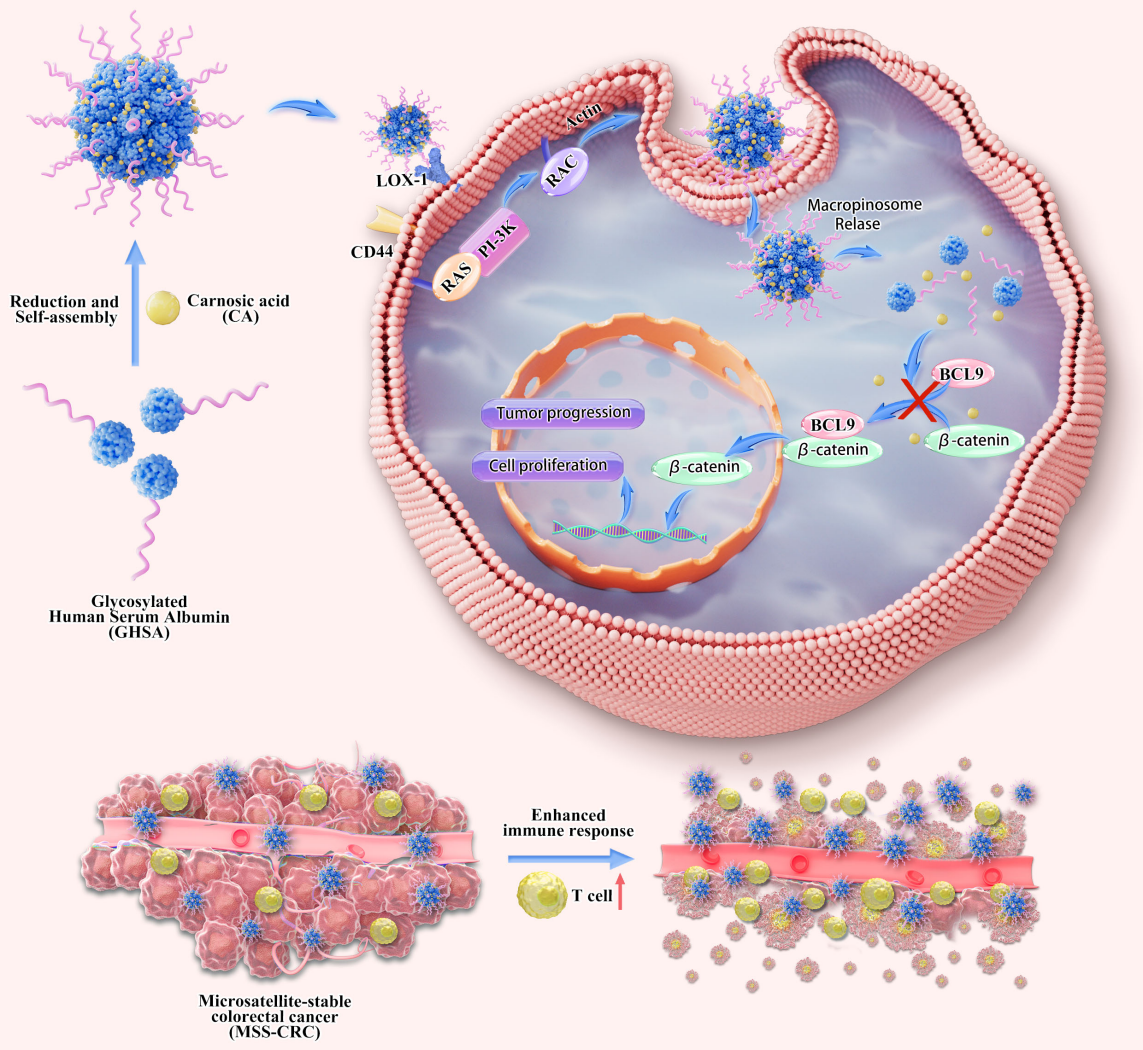
Illustrates the synthesis, endocytosis, and mechanism of GHSACA, which efficiently targets delivery through the LOX1 receptor-mediated macropinocytosis pathway. Upon reaching tumor tissues, GHSACA releases CA within tumor cells, which disrupts β-catenin/BCL9 binding, downregulates oncogenic Wnt signaling, modulates the immunosuppressive microenvironment, and suppresses tumor growth.

This study aims to elucidate the mechanism of action of GHSACA nanomedicine and its potential application in treating MSS-CRC. Previous research indicates that GHSACA is internalized into tumor cells via the macropinocytosis pathway. We discovered that GHSACA primarily enters tumor cells via the LOX1-mediated macropinocytosis pathway. In patient-derived xenograft (PDX) models, we systematically assessed the anti-tumor efficacy of GHSACA both as a monotherapy and in combination with PD-1 inhibitors. The findings demonstrated that GHSACA effectively inhibited the Wnt/β-catenin pathway, reduced downstream oncogene expression, and significantly increased cytotoxic T cell infiltration and activity in the tumor microenvironment. The combination of GHSACA and PD-1 inhibitors exhibited a strong synergistic effect, presenting a promising new therapeutic strategy to overcome immunotherapy resistance in MSS-CRC (**Figure 1**).

## 2 Materials and methods

### 2.1 Experimental materials

MES, NHS, EDC and HAuCl_4_·xH_2_O were procured from Aladdin Chemicals. Human serum albumin and glucosamine were obtained from SAITONG and RHAWN, respectively. Small interfering RNAs (siRNAs) targeting LOX1 and CD44, as well as control siRNAs, were purchased from Tsingke Biotechnology Co. Ltd. Chemicals for this study were sourced from Sigma Aldrich unless noted otherwise, and were used as received without additional purification.

### 2.2 Preparation of GHSA

To activate the carboxyl groups of HSA, EDC (100 mM) and NHS (200 mM) were added to Solution 1, an activation buffer with 13.2 mg/mL human serum albumin in 0.1 M MES and 0.5 M NaCl at pH 6.0, and incubated at room temperature for 15 minutes. The pH was then adjusted to above 7.0 using 10×phosphate-buffered saline (PBS). A PBS solution with 3.6 mg/mL glucosamine was added to the activated HSA solution, and the mixture was incubated at room temperature for 2 hours to promote glycation. Post-conjugation, the product was purified via 24-hour dialysis against deionized water, employing a regenerated cellulose membrane with a 3,000 Da molecular weight cutoff. The dialyzed solution was collected and lyophilized to yield the final GHSA conjugate as a lyophilized powder.

### 2.3 Synthesis of GHSACA

5 mg of GHSA protein was dissolved in 500 μL of PBS (pH 7.4), and 500 μL of tris (2-carboxyethyl) phosphine (TCEP,1 mg/mL) was added. After magnetic stirring for 10 minutes to achieve complete reduction of disulfide bonds,3.5 mL of PBS was added immediately to dilute the reaction mixture. The solution was then mixed with a pre-prepared solution containing 10μL of dimethyl sulfoxide (DMSO) and 5 mg of CA, and the resulting mixture was subjected to ultrasonication for 20 minutes to facilitate conjugation. Subsequently, 500 μL of a 10 mM aqueous solution of tetrachloroauric acid (HAuCl_4_) was added dropwise under gentle stirring. The reaction continued at room temperature with constant magnetic stirring for 10 minutes to facilitate gold nanoparticle formation and conjugation. Unreacted reagents and low-molecular-weight byproducts were eliminated through dialysis with a 10 kDa cutoff regenerated cellulose membrane, followed by two distilled water washes. The purified product was collected and designated as the nanomedicine GHSACA.

### 2.4 *In vitro* experiments

#### 2.4.1 Cell culture

The murine colon cancer cell lines MC38 and CT26 were obtained from the Shanghai Cell Bank of the Chinese Academy of Sciences. Cells were cultured in RPMI-1640 medium (Gibco, Thermo Fisher Scientific) with 10% fetal bovine serum and 1% penicillin-streptomycin. Cultures were incubated at 37 °C with 5% CO_2_ and the medium was renewed every 2–3 days to maintain optimal growth conditions.

#### 2.4.2 Transient siRNA transfection

To explore the roles of LOX1 and CD44 receptors in drug uptake, siRNAs targeting LOX1 and CD44, along with a negative control siRNA, were procured from Tsingke Biotechnology Co.Ltd.si-LOX1 has the sequence GCAACUGUGCAUACCUUCAdTdT (sense) and UGAAGGUAUGCACA GUUGCdTdT (antisense). si-CD44 has the sequence GGCUUUCAACAGUACCUUAdTdT (sense) and UAAGGUACUGUUGAAAGCCdTdT (antisense). MC38 and CT26 cells were seeded (250,000 cells/well) on six-well culture dishes to 60-70% confluence. One hour before transfection, the culture medium was replaced with a serum-free and antibiotic-free medium. Following the manufacturer’s guidelines, cells were incubated for 5 hours with transfection mixtures containing 100 nmol of si-LOX1,si-CD44 or si-NC using Lipofectamine 2,000. Subsequently, the medium was changed to a complete culture medium. After an additional incubation period of 24–72 hours, cells reaching approximately 90% confluence were collected for further analysis.

#### 2.4.3 Validation of gene knockdown efficiency by RT-qPCR

RNA extraction from each experimental group’s cells was performed using TRIzol reagent following the manufacturer’s instructions. RNA concentration and purity were evaluated with a NanoDrop spectrophotometer to confirm A260/A280 ratios within the acceptable range of 1.8 to 2.0.A total of 1 μg RNA was reverse- transcribed into cDNA using the PrimeScript RT Reagent Kit with gDNA Eraser to remove genomic DNA contamination. The cDNA served as a template for qPCR amplification using the SYBR Green Premix Pro Taq HS qPCR Kit on a QuantStudio 5 Real-Time PCR System. Amplification was carried out under standardized thermal cycling conditions, and gene-specific primers for LOX1 and CD44 were used. GAPDH was selected as the endogenous reference gene for normalization. The 2^−ΔΔCt method was employed to calculate relative mRNA expression levels. All experiments were performed in biological triplicate and technical duplicate to ensure reproducibility and statistical reliability.

#### 2.4.4 Cellular uptake assay of FITC-labeled GHSACA

MC38 and CT26 cell lines, including wild-type (WT), negative control siRNA (si-NC), LOX1-knockdown (si-LOX1), CD44-knockdown (si-CD44), and dual LOX1/CD44-knockdown (si-LOX1/si-CD44), were seeded into 6-well plates at suitable densities and cultured to approximately 50% confluence. The medium was refreshed with growth medium containing 10 μg/mL FITC-labeled GHSACA in PBS (pH 7.4). Cells were incubated at 37 °C for 6 hours to facilitate drug uptake. After incubation, cells were washed thrice with ice-cold PBS to eliminate non-internalized nanoparticles and then resuspended in PBS. Samples were immediately analyzed by flow cytometry to quantify intracellular fluorescence intensity as a measure of cellular drug uptake.

#### 2.4.5 Dextran uptake assay

To evaluate the functional activity of LOX1 and CD44 receptors, specific ligand uptake assays were performed. Dextran Uptake Assay for LOX1 and CD44 Functional Assessment: Cells from each experimental group were seeded in 6-well plates and cultured to approximately 50% confluence. 2 mL of RPMI-1640 complete medium, pre-warmed and supplemented with 1 mg/mL dextran, was added to each well. Cells were then incubated at 37 °C for 2 hours to facilitate dextran internalization. Following incubation, cells underwent three washes with ice-cold PBS to eliminate surface-bound dextran. The percentage of dextran positive cells was then determined by flow cytometry.

### 2.5 *In vivo* experiments

BALB/c and NOD/SCID mice for this study were sourced from Xi’an Jiaotong University’s experimental animal center. Animal experiments were performed following guidelines approved by the Xi’an Jiaotong University Medical Ethics Committee, protocol number 2022-1104. The study complied with both national and institutional guidelines for laboratory animal care and use. Mice were anesthetized via isoflurane inhalation (2-5% for induction in a closed chamber, 2-3% for maintenance with a nose cone) until loss of righting reflex. At the end of the experiment, euthanasia was performed with an overdose of sodium pentobarbital (100 mg/kg, intraperitoneal injection), and death was confirmed by the absence of respiration, heartbeat, and reflexes for ≥5 minutes.

#### 2.5.1 *In vivo* fluorescence imaging

BALB/c mice with CT26 cells received a 200 μL tail vein injection of Cy5- labeled GHSACA (1 mg/mL). Euthanasia occurred at intervals of 0, 2, 4, 8, 12, 24 and 48 hours post-administration, followed by the excision of major organs (heart, liver, spleen, lung, kidney) and tumor tissues for ex vivo fluorescence imaging and semi- quantitative analysis.

#### 2.5.2 Establishment of MSS-CRC PDX Model in NOD/SCID mice

Tumor tissues obtained from patients with advanced MSS-CRC were subcutaneously implanted into 5-week-old female NOD/SCID mice to generate the primary passage (PDX-P0). Approximately two months later, when tumor volumes reached approximately 1,000 mm³, P0 tumors were harvested and serially passaged into NSG mice to establish the PDX-P1 generation. Using the same engraftment protocol, P1-derived tumors were further passaged to generate the PDX-P2 cohort, which was used for subsequent formal experiments. After four weeks of growth, when tumor volumes reached 50–100 mm³, mice were randomly divided into five groups (n = 5 per group): control (PBS), GHSA, anti-PD-1 antibody, GHSACA, and combination therapy (GHSACA+anti-PD-1 antibody, G&P). On day 0,all mice received an intravenous injection of 750,000 human peripheral blood mononuclear cells (PBMCs) in 200 μL via the tail vein. Beginning on day 1, treatments were intravenously administered via the tail vein on days 1, 3, 5, 7, 9 and 11 with a consistent dose of 3 mg/kg in a total volume of 200 μL. An additional PBMC injection was given on day 6 to support sustained immune cell engraftment. Body weight and tumor volume were assessed bi-daily throughout the treatment period. Tumor growth inhibition (TGI) was determined using the formula: TGI = [1−(RTV_ treatment/RTV _control)]×100%, where RTV (relative tumor volume) is calculated as the final tumor volume (Vt) divided by the initial tumor volume (V_0_). Following ethical guidelines, mice were humanely euthanized at the study’s conclusion, and tumor tissues were collected for *in vivo* analysis.

#### 2.5.3 Immunohistochemical staining

PDX tumor tissues, preserved in formalin and embedded in paraffin, were sectioned into 5 μm slices with a microtome and placed on glass slides. Sections were deparaffinized using xylene and rehydrated through a series of graded ethanol. Endogenous peroxidase activity was inhibited by treating with 3% hydrogen peroxide for 30 minutes at room temperature. Antigen retrieval involved a 10-minute high- pressure steam treatment using Tris-EDTA buffer at pH 9.0.Following cooling to room temperature, the sections were incubated overnight at 4 °C with primary antibodies against β-catenin (Abcam, ab32572), C-myc (Abcam, ab32072), Cyclin D1 (CST, 55506), Ki-67 (CST, 12202T), PD-L1 (Abcam, ab205921) and Granzyme B (Abcam, ab255598). The next day, sections were incubated with HRP-conjugated secondary antibodies for 1 hour at room temperature. Immunoreactivity was visualized using a 3,3′-diaminobenzidine (DAB) chromogenic detection kit. Sections were counterstained with hematoxylin, dehydrated, cleared in xylene, and permanently mounted using neutral balsam. All staining procedures were performed under standardized conditions with negative controls included to ensure specificity. Scanning and digital imaging were performed using Slide Viewer software (3DHISTECH). Five randomly selected high-power fields (400× magnification) from each tissue section were analyzed to quantify the average optical density or count of positively stained cells. Tumor cell apoptosis was evaluated using the TUNEL detection kit (Roche Diagnostics) following the provided protocol.

#### 2.5.4 Immunofluorescence double staining for CD3^+^/CD8^+^ and CD4^+^/CD25^+^ T cells

Primary antibody mixtures included anti-CD3 (Proteintech, 17617-1-AP) with anti-CD8 (Abcam, ab22378), or anti-CD4 (Abcam, ab183685) with anti-CD25 (Abcam, ab128955). Sections were incubated overnight at 4 °C, then treated with secondary antibodies: Alexa Fluor 594-conjugated goat anti-mouse IgG and Alexa Fluor 488-conjugated goat anti-rabbit IgG. Fluorescence microscopy was used to manually quantify CD3^+^/CD8^+^ (cytotoxic T cells) and CD4^+^/CD25^+^ (regulatory T cells phenotype) double-positive cells in five randomly selected high-power fields (400×magnification) per section.

### 2.6 Statistical analysis

GraphPad Prism software (version 8.0) was used for statistical analyses. Group comparisons were conducted using a two-tailed unpaired Student’s t-test for two-group analyses or a one-way ANOVA with Tukey’s *post hoc* test for multiple groups, depending on the experimental design. Quantitative data are expressed as mean ± standard deviation (SD). Statistical significance was determined by a P-value below 0.05.

## 3 Results

### 3.1 LOX1/CD44-mediated internalization of GHSACA and activation of macropinocytosis

**Figure 2:**
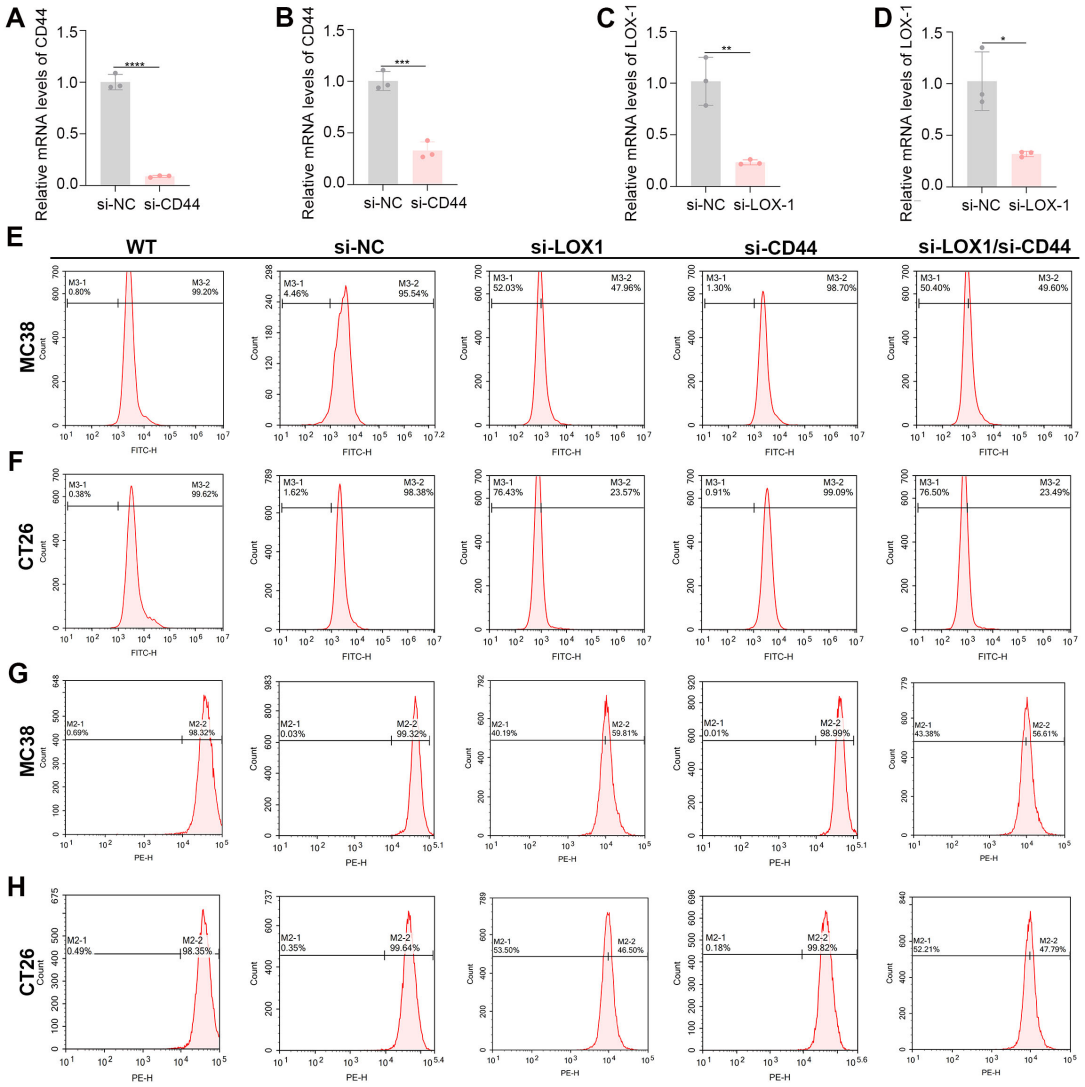
Validation of LOX1 and CD44 knockdown, flow cytometric analysis of the regulation of GHSACA internalization and macropinocytic activity in cells. **(A)** CD44 expression in MC38 cells after transfection with si-NC or si-CD44. **(B)** CD44 expression in CT26 cells after transfection with si-NC or si-CD44. **(C)** LOX1 expression in MC38 cells after transfection with si-NC or si-LOX1. **(D)** LOX1 expression in CT26 cells after transfection with si-NC or si-LOX1. **(E, F)** Uptake efficiency of FITC-GHSACA in MC38 and CT26 cells with different gene knockdowns. **(G, H)** Uptake efficiency of dextran in MC38 and CT26 cells under distinct gene knockdown conditions (macropinocytosis activity assay). * = p < 0.05 ** = p < 0.01 *** = p < 0.001 **** = p < 0.0001.

Following siRNA-mediated knockdown of LOX1 and CD44 in murine colon cancer cell lines MC38 and CT26, gene expression levels were evaluated by RT-qPCR. The findings indicated a notable decrease in target mRNA expression in the si-LOX1 and si-CD44 groups relative to the si-NC group (p<0.01), validating the successful silencing of LOX1 and CD44 in both cell lines (**Figures 2A–D**).

Flow cytometry analysis indicates that LOX1 is the main receptor facilitating GHSACA internalization and activation of the macropinocytosis pathway, while CD44 plays a negligible role. In drug uptake assays, LOX1 knockdown significantly decreased FITC-GHSACA internalization, with FITC-positive MC38 cells dropping to 47.96% in the si-LOX1 group compared to 95.54% in the si-NC control group, and CT26 cells showing a reduction to 23.57% in the si-LOX1 group versus 98.38% in the si-NC group. Notably, dual knockdown of LOX1 and CD44 (23.49%) showed no significant difference compared to the si-LOX1 single-knockdown group, further supporting the predominant role of LOX1 in mediating cellular uptake. In contrast, CD44 knockdown had minimal impact on drug internalization: the FITC-positive rates in the si-CD44 groups were 98.7% and 99.09% in MC38 and CT26 cells, respectively (**Figures 2E, F**).

To clarify the mechanism, cellular uptake of dextran, a macropinocytosis substrate, was assessed. LOX1 knockdown significantly reduced macropinocytic activity, with dextran-positive MC38 cells at 59.81% in the si-LOX1 group compared to 99.32% in the si-NC group. In CT26 cells, the positive rate dropped to 46.5% in the si-LOX1 group, much lower than the 99.64% in the si-NC group. Similarly, the dual knockdown group (47.79%) exhibited a comparable reduction to the si-LOX1 single-knockdown group, indicating that LOX1 functions as a central regulator of the macropinocytosis pathway (**Figures 2G, H**). CD44 knockdown did not significantly impact dextran internalization, as the positive rates in the si-CD44 groups were 98.99% for MC38 cells and 99.82% for CT26 cells, showing no statistical difference from the si-NC group. Collectively, these findings define the functional cascade”LOX1– macropinocytosis–GHSACA internalization” and confirm that GHSACA is internalized into colorectal cancer cells predominantly via the LOX1-mediated macropinocytosis pathway, with CD44 playing no discernible role in this process.

### 3.2 *In vivo* biodistribution of GHSACA and its anti-tumor activity in MSS-CRC PDX model

**Figure 3:**
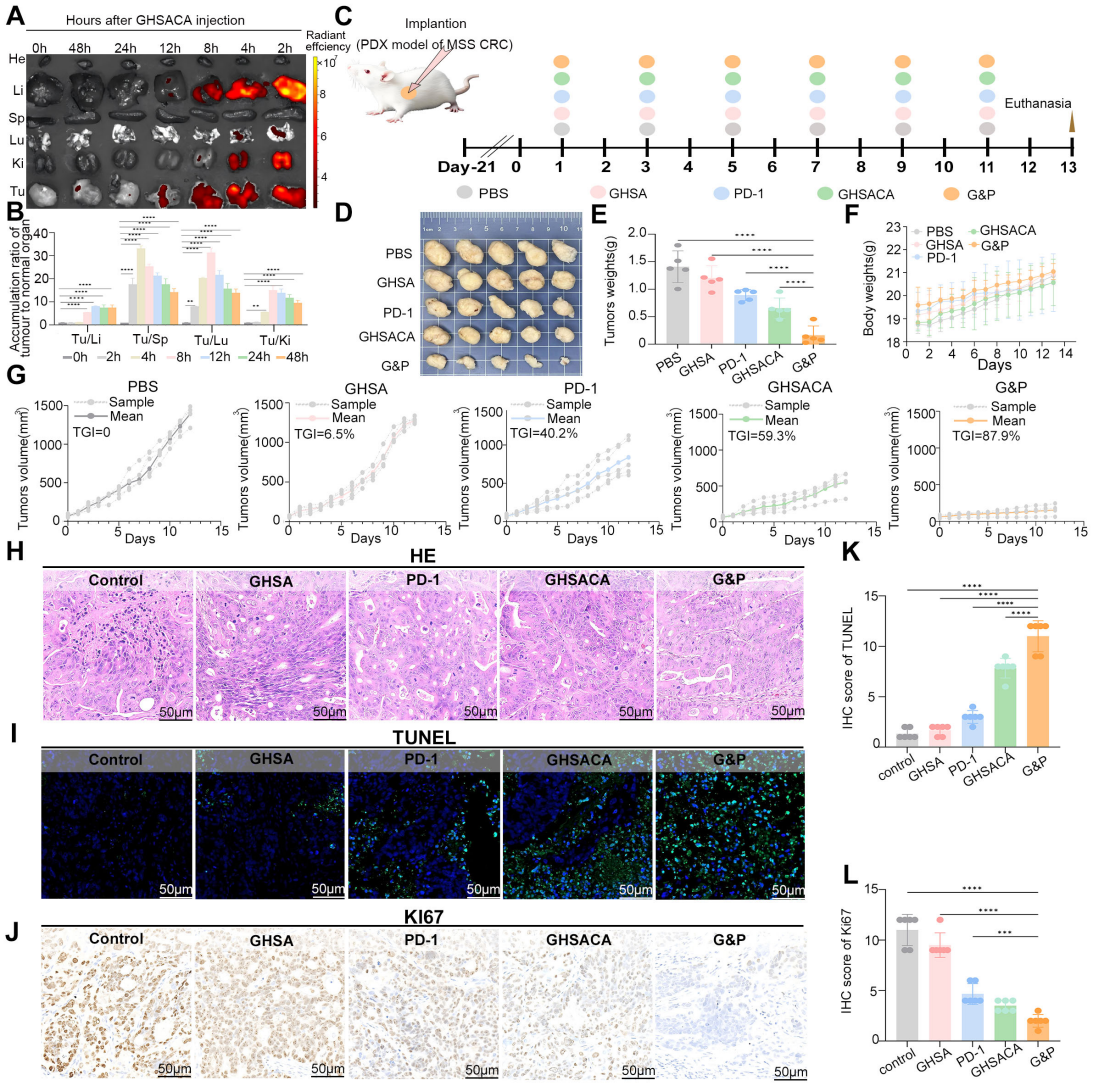
*In vivo* fluorescence imaging analysis of GHSACA distribution and the antitumor efficacy in MSS-CRC PDX model. **(A)** Fluorescence distribution images of GHSACA in major organs: Heart (He), Liver (Li), Spleen (Sp), Lung (Lu), Kidney **(Ki)** and tumor tissues (Tu) from CT26 tumor-bearing BALB/c mice. **(B)** Quantitative comparison of GHSACA accumulation in tumor tissues versus normal organs. **(C)** Schematic diagram of the MSS-CRC-PDX model implantation, corresponding treatment and sample analysis in NOD/SCID mice. **(D)** Macroscopic morphological comparison of tumor tissues from PDX models in various treatment groups. **(E)** Statistical analysis of tumor weight in PDX models among different treatment groups. **(F)** Body weight variations over time in PDX-bearing mice across different treatment groups. **(G)** Tumor volume dynamics in PDX models under various treatment regimens. **(H)** Representative H&E staining images of tumor sections with the indicated treatments. **(I)** Representative TUNEL staining images of tumor sections under different treatments and **(K)** the IHC score. **(J)** IHC staining for Ki67 and **(L)** the IHC score. ** = p < 0.01 *** = p < 0.001**** = p < 0.0001.

*In vivo* fluorescence imaging assessed the biodistribution of Cy3PMI-labeled GHSACA in both tumor and normal mouse tissues. Our study found that following administration, the agent achieved peak concentrations in key organs including the liver, lung, and kidney within 2 hours. Subsequently, the agent was nearly undetectable in the lung at 4 hours, kidney at 8 hours, and liver at 12 hours post- administration, which was attributed to its rapid clearance from normal organs. In contrast, tumor tissues (Tu) exhibited sustained and selective retention of the drug, with high fluorescence signals maintained up to 48 hours before a gradual decline was observed. The results demonstrate that GHSACA exhibits favorable tumor-targeting properties *in vivo*, effectively minimizing damage to normal tissues and establishing a foundation for subsequent *in vivo* efficacy evaluations. Quantitative analysis of the drug accumulation ratio between tumor and normal tissues revealed significantly higher levels in tumor sites across all observed time points (2 h, 4 h, 8 h, 12 h, 24 h, and 48 h). These findings further substantiate the superior tumor-targeting capability of GHSACA, indicating its selective enrichment at tumor sites and reduced impact on normal tissues (**Figures 3A, B**).

In the PDX model, tumor cells were implanted 21 days prior to the initiation of treatment. Beginning on day 0, mice received treatments every other day for about 13 days, including PBS (blank control), GHSA, anti-PD-1 antibody (PD-1), GHSACA or a combination of GHSACA and anti-PD-1 (G&P). Following this period, the mice were euthanized for subsequent tumor-related analyses (**Figure 3C**). Post-sacrifice, tumor tissues were excised and photographed for morphological analysis (**Figure 3D**). Statistical assessment of tumor weights revealed that, relative to the PBS control group, the GHSA group had a slight reduction, the PD-1 group experienced a more notable decrease, the GHSACA group showed a significantly larger reduction, and the G&P combination group exhibited the greatest decrease in tumor weight (**Figure 3E**). Further analysis of mouse body weight revealed consistent temporal trends across all treatment groups with no significant differences, indicating that neither GHSACA, anti-PD-1 monotherapy, nor their combination induced marked systemic toxicity, thus demonstrating favorable safety profiles (**Figure 3F**). To evaluate antitumor efficacy across treatment groups, the TGI was calculated (**Figure 3G**). The PBS control group exhibited a TGI of 0, with tumors following a natural growth trajectory and showing progressive volume expansion over time. The GHSA group demonstrated a TGI of 6.5%, indicating minimal antitumor activity and only modest reduction in tumor growth. In the PD-1 group, a TGI of 40.2% was observed, accompanied by markedly slower tumor progression compared to both the PBS and GHSA groups. The GHSACA group achieved a TGI of 59.3%, with further suppression of tumor growth and significant tumor volume reduction. Notably, the G&P combination group showed the highest TGI of 87.9%, with near-complete inhibition of tumor growth, representing the most robust antitumor efficacy among all treatment regimens.

Hematoxylin-eosin (HE) staining of PDX tumor tissue sections was performed to evaluate tissue morphology. In the Control group, tissue cells exhibited irregular morphology and disorganized arrangement, indicative of a higher prevalence of aberrant cellular phenotypes. In contrast, the GHSACA group exhibited distinct morphological characteristics, including unique cellular structures and reorganized tissue patterning compared to other treatment groups. Notably, the G&P combination group demonstrated the most pronounced morphological remodeling, indicating that the combined regimen exerts substantial effects on tissue microstructure (**Figure 3H**). TUNEL staining showed that the Control group exhibited only a limited number of TUNEL-positive cells, indicating a low basal level of apoptosis. Compared with Group GHSA and PD-1 monotherapy group, the GHSACA group exhibited a marked increase in TUNEL-positive cells, confirming its effectiveness in promoting apoptosis. The G&P combination group exhibited the highest density of apoptotic cells, indicating a strong induction of apoptosis and suggesting a synergistic mechanism for enhanced apoptotic activity (**Figures 3I,K**). Immunohistochemical analysis showed a significant reduction in Ki67-positive cells and staining intensity, highlighting its ability to substantially inhibit tumor cell proliferation (**Figures 3J, L**).

### 3.3 The G&P combination therapy synergistically enhances antitumor effects by simultaneously inhibiting the Wnt/β-catenin pathway and boosting antitumor immunity

**Figure 4:**
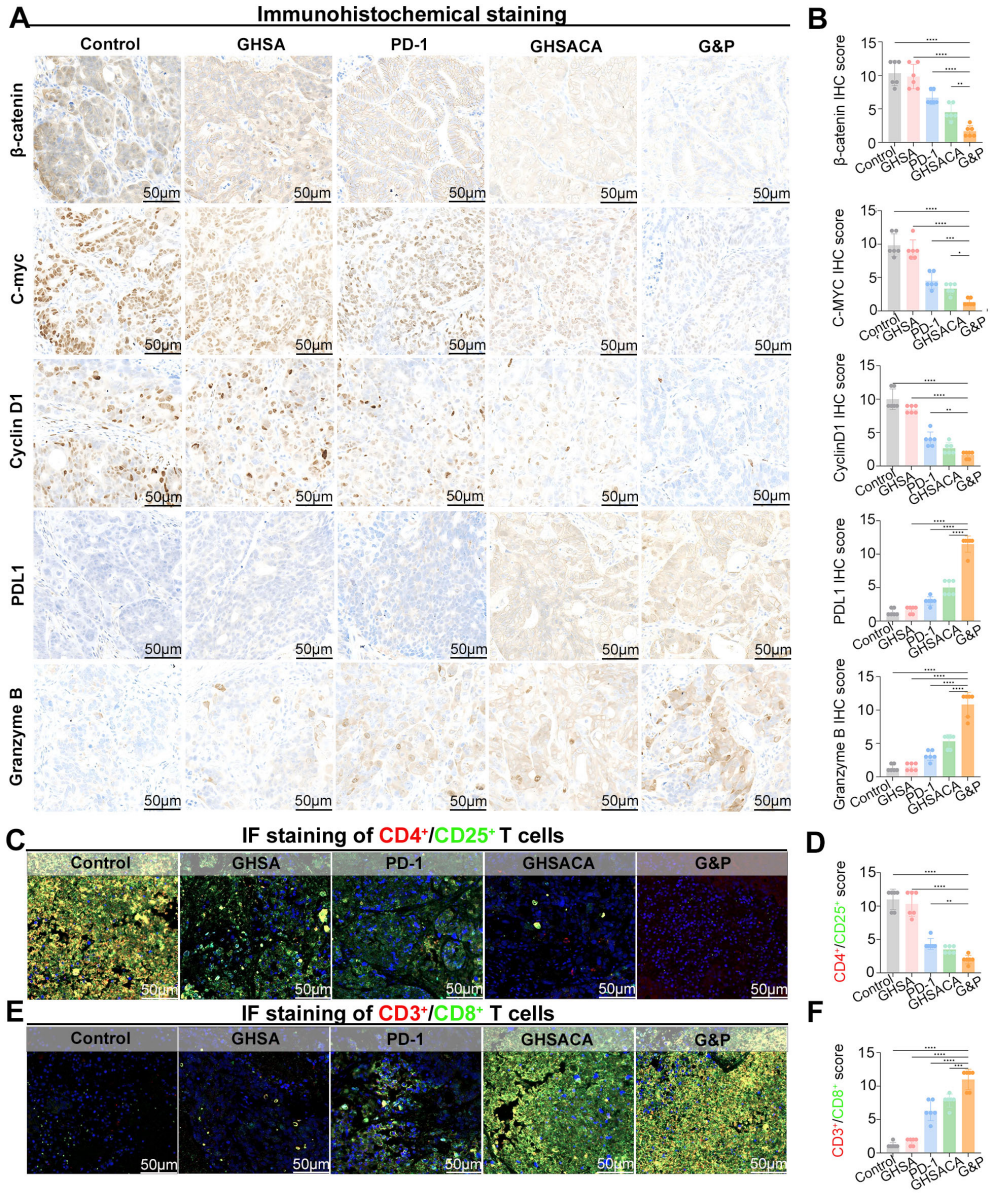
Immunohistochemical **(IHC)** staining of tumor tissues and Immunofluorescence double-staining analysis of T-cell subset infiltration from MSS-CRC PDX models. **(A)** IHC staining was performed for β-catenin, C-myc, Cyclin D1, PD-L1, and Granzyme **(B) (B)** IHC scores for β- catenin, C-myc, Cyclin D1, PD-L1, and Granzyme **(B) (C)** Immunofluo-rescence images showing CD4^+^/CD25^+^ regulatory T-cell infiltration in tumor tissues. **(D)** IHC score of CD4^+^/CD25^+^ T cells. **(E)** Immunofluorescence images showing CD3^+^/CD8^+^ cytotoxic T-cell infiltration in tumor tissues. **(F)** IHC score of CD3^+^/CD8^+^ T cells. * = p < 0.05 ** = p < 0.01 *** = p < 0.001 **** = p < 0.0001.

To investigate GHSACA’s anti-tumor activity and its synergistic immunotherapy mechanism, we performed immunohistochemical analysis on tumor tissue sections from each group. Tumor tissues from the Control and GHSA groups showed prominent β-catenin reactivity. The PD-1 group had moderate downregulation of β-catenin expression, while the GHSACA and G&P groups exhibited significant reductions in β-catenin-positive staining. Quantitative analysis revealed significantly reduced IHC scores in the two groups, demonstrating effective inhibition of aberrant Wnt/β-catenin signaling. This inhibition is due to GHSACA’s ability to disrupt the β-catenin–BCL9 interaction, preventing β-catenin’s nuclear translocation and transcriptional activation. Consequently, expression levels of key Wnt/β-catenin downstream oncogenes, such as C-myc and Cyclin D1, were significantly lower in the GHSACA treatment groups, with the combination therapy group showing a more pronounced reduction (**Figures 4A, B**).

In order to explore the specific mechanism by which GHSACA can enhancing antitumor immunity, we conducted further research. Distinct regulatory patterns of PD-L1 expression were observed across treatment groups. Both the Control and GHSA groups showed low baseline PD-L1 expression in tumor and tumor-infiltrating immune cells. The PD-1 group exhibited a slight rise in PD-L1 expression, while GHSACA monotherapy led to further upregulation. The G&P combination therapy produced the highest increase in PD-L1 expression. Importantly, the G&P group showed a significant presence of densely distributed Granzyme B-positive granules across the tumor tissue. The IHC score for this group was notably higher compared to all other treatment groups (**Figures 4A, B**). Immunofluorescence staining of cancer tissues showed reduced CD4^+^/CD25^+^ T cell infiltration and significantly increased CD3^+^/CD8^+^ T cell infiltration in the G&P group compared to the other groups (**Figures 4C–F**). This suggests that G&P combination therapy synergistically enhances CD8^+^ T cell infiltration while reducing immunosuppressive regulatory T cell (Treg) accumulation in the MSS-CRC PDX model.

### 3.4 Systematic evaluation of major organ and hematopoietic system toxicity

**Figure 5:**
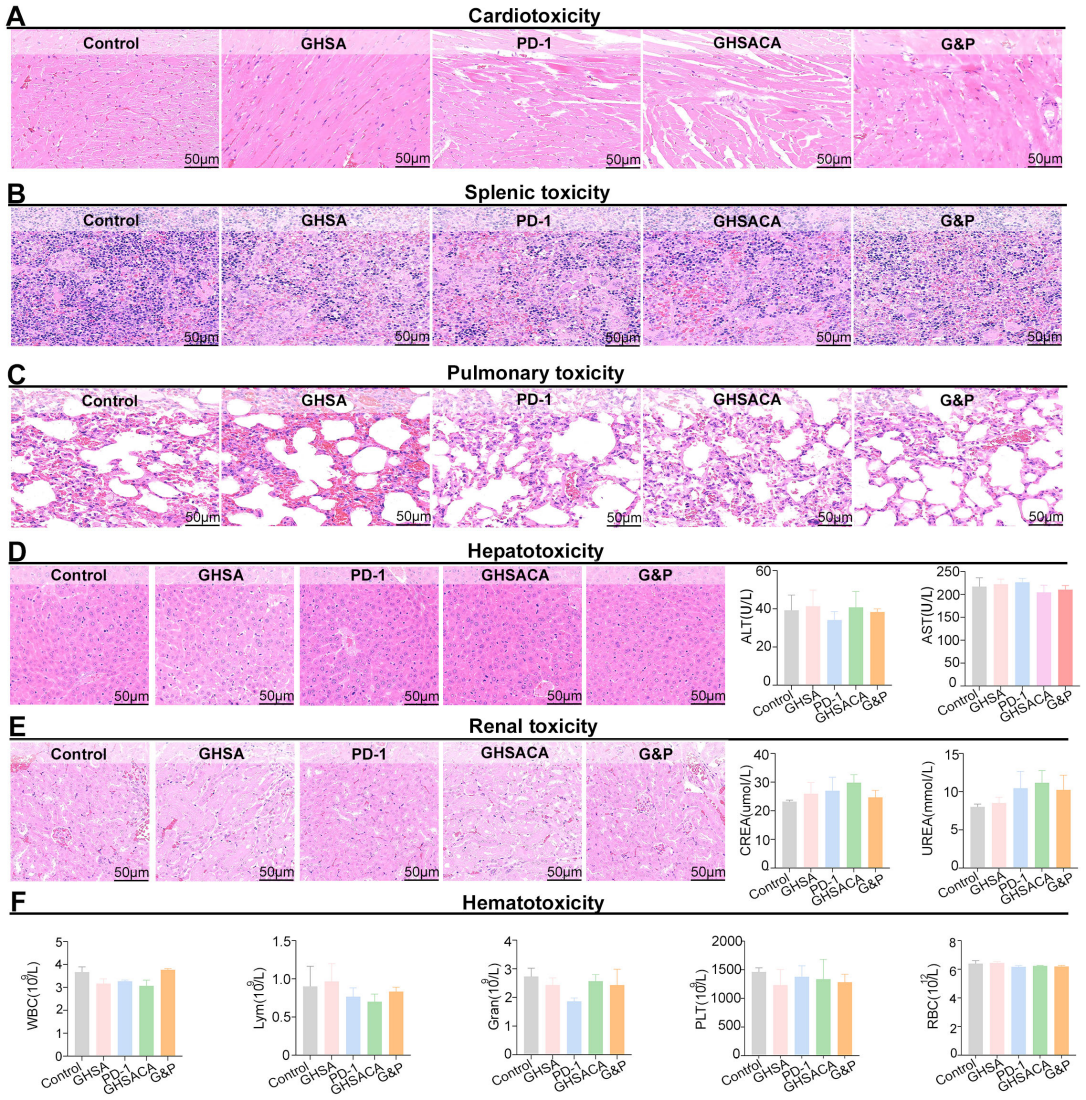
Safety evaluation of GHSACA monotherapy and combination therapy with PD-1 antibody. **(A)** Histological examination of cardiac tissues from each treatment group using H&E staining. **(B)** Histological examination of splenic tissues using H&E staining. **(C)** Pulmonary tissue sections were stained using H&E. **(D)** H&E staining of hepatic tissues and hepatic biochemistry. **(E)** Histological analysis using H&E staining and renal biochemistry assessment of kidney tissues. **(F)** Statistical analysis of key hematological parameters in peripheral blood.

Histopathological analysis showed no significant pathological changes in myocardial tissues among the GHSA, PD-1, GHSACA, and G&P treatment groups compared to the Control group. Specifically, no inflammatory cell infiltration, myofiber necrosis, or interstitial edema was observed. These findings indicate that none of the experimental therapeutic regimens induced marked cardiotoxicity during the study period (**Figure 5A**). Histopathological examination of splenic tissues showed preserved architectural integrity in all treatment groups, with both white pulp and red pulp maintaining normal structural organization. Compared with the Control group, no pathological abnormalities—such as splenic atrophy, necrosis, or hyperplastic changes—were detected in any of the experimental groups. The findings indicate that the administered therapies did not cause significant toxic effects on the spleen (**Figure 5B**). Morphological analysis showed that lung tissues across all treatment groups maintained well-preserved alveolar structures with intact septa, without signs of interstitial thickening, inflammatory cell infiltration, or alveolar exudate. The histopathological characteristics were similar to the Control group, suggesting no significant pulmonary injury from the treatments (**Figure 5C**). Hematoxylin and eosin (H&E) stained liver sections revealed no evidence of inflammatory cell infiltration or hepatic necrosis. Biochemical serum tests showed normal alanine aminotransferase (ALT) and aspartate aminotransferase (AST) levels across all groups, with no significant differences, indicating an absence of hepatotoxicity. (**Figure 5D**). Histopathological evaluation of renal tissues revealed preserved glomerular and tubular architectures in all treatment groups, with no evidence of glomerular atrophy, sclerosis, tubular epithelial cell swelling, degeneration, or cast formation. Compared with the Control group, no drug-related pathological alterations were observed. Meanwhile, creatinine (CREA) and UREA levels were consistent among all groups, indicating a favorable renal safety profile of the experimental regimen (**Figure 5E**). Hematotoxicity assessment showed that all key hematological parameters in the treatment groups remained within normal physiological ranges relative to the Control group, with no features indicative of typical myelotoxicity or sustained suppression of any specific cell lineage (**Figure 5F**).

In summary, none of the treatments—GHSA, PD-1 monotherapy, GHSACA, or the G&P combination therapy—caused significant pathological damage to vital organs such as the heart, spleen, lung, liver, or kidney. Furthermore, hematological analysis revealed no evidence of severe myelosuppressive toxicity. These findings provide preliminary yet essential supportive evidence for the safety profile of these therapeutic regimens, particularly the G&P combination therapy, in subsequent preclinical or clinical investigations.

## 4 Discussion

This study reports the successful design and synthesis of a novel glycosylated human serum albumin-based nanomedicine, GHSACA. Systematic investigations revealed that GHSACA efficiently targets delivery through the LOX1 receptor- mediated macropinocytosis pathway. In the MSS-CRC PDX model, GHSACA exhibits significant therapeutic efficacy by concurrently inhibiting the Wnt/β-catenin signaling pathway and activating the anti-tumor immune response. Particular importantly, the combination therapy of GHSACA and PD-1 inhibitors (G&P) elicits a potent synergistic effect. This finding offers a novel strategy with high translational potential for overcoming the immunotherapy resistance in MSS-CRC.

The targeted delivery efficiency of nanomedicines constitutes a fundamental determinant of their therapeutic efficacy. Surprisingly, our findings demonstrate that LOX1 rather than the commonly assumed CD44 receptor, is the primary mediator of GHSACA cellular internalization (10). LOX1, a scavenger receptor, is highly expressed on tumor vascular endothelial cells and various cancer cell types, with its expression level positively correlated with tumor malignancy (11, 12). Experimental results show that LOX1 knockdown markedly reduces both GHSACA uptake and macropinocytic activity, whereas CD44 knockdown exerts no significant effect. This finding has dual implications. First, it reveals a distinct targeting mechanism for GHSACA: the nanoparticle exploits the overexpression of LOX1 on tumor cells and their heightened macropinocytic activity to achieve selective intratumoral accumulation. This mechanism is consistent with the pronounced tumor-targeting specificity and favorable pharmacokinetic profile—characterized by rapid systemic clearance—observed *in vivo (*13). Second, macropinocytosis serves as a critical pathway for nutrient acquisition in tumor cells, particularly under oncogenic Ras activation or metabolic stress conditions (14). By hijacking this endocytic process through a “Trojan-horse” strategy, GHSACA may maintain efficient delivery even within the hypoxic and nutrient-deprived tumor core, regions typically resistant to conventional drug penetration. This mechanism provides a plausible explanation for the robust antitumor efficacy of GHSACA *in vivo (*15). Although the dominant role of LOX1 over CD44 in GHSACA internalization has been well-documented, the structural and functional underpinnings of this receptor preference remain to be fully elucidated. Such investigations will provide critical insights for the rational design of advanced nanocarrier systems in future studies.

In PDX models of MSS-CRC, GHSACA administered as a monotherapy demonstrated significant tumor growth inhibition. Its therapeutic efficacy markedly surpassed that of PD-1 inhibitor monotherapy, highlighting the pivotal role of targeting core oncogenic signaling pathways in the treatment of MSS-CRC (16). The underlying mechanism stems from the effective suppression of aberrant Wnt/β-catenin pathway activation by CA delivered via GHSACA. This was demonstrated by decreased accumulation of β-catenin protein in both the cytoplasm and nucleus, as well as the transcriptional downregulation of key target genes such as C-myc and Cyclin D1. These molecular changes led to direct antitumor effects, including cell cycle arrest (via Cyclin D1 downregulation), inhibition of cellular proliferation (as indicated by decreased Ki67 expression), and induction of apoptosis (confirmed by TUNEL assay). Our findings align with existing literature showing that inhibiting the Wnt/β-catenin pathway can mitigate its immunosuppressive effects and reverse immune exclusion in the tumor microenvironment (17, 18). In this study, treatment with GHSACA resulted in a marked increase in granzyme B–positive cells and enhanced infiltration of CD8^+^ T cells within the tumor. These immunological changes demonstrate that GHSACA successfully converts immunologically “cold” tumors into “hot” tumors with increased immune activity, fostering a more favorable microenvironment for further immunotherapy (9, 19).

GHSACA mitigated the inhibition of T-cell chemokines like CCL4 by blocking the β-catenin pathway. This enhanced the repertoire of T cells available for targeting by the PD-1 antibody (6, 20). Conversely, the PD-1 antibody relieved the functional inhibition of these T cells, enabling them to efficiently kill tumor cells (21). The G&P group exhibited the highest Granzyme B expression and the densest CD8^+^ T cell infiltration. Notably, the G&P combination therapy also led to a significant decrease of CD4^+^/CD25^+^ regulatory T cells (Tregs) within tumors. This is likely a compensatory feedback mechanism triggered by strong anti-tumor immunity, aiming to maintain immune homeostasis (22, 23). Although this may potentially limit the durability of treatment efficacy, it precisely points to the direction for optimizing future combination strategies. Incorporating treatments that target or inhibit Tregs, like anti-CTLA-4 antibodies, could enhance therapeutic outcomes. Notably, the combination treatment group showed a marked increase in PD-L1 expression. This is generally regarded as an “adaptive immune resistance” response of tumor cells and myeloid-derived suppressor cells to immune pressures such as IFN-γ (24, 25). The upregulation of PD-L1 in the presence of a PD-1 inhibitor may enhance tumor cell sensitivity to immune checkpoint blockade, potentially explaining the superior therapeutic efficacy observed in the G&P group (26, 27). This finding indicates that PD-L1 levels in tumors treated with GHSACA could be a potential biomarker for predicting the effectiveness of combination therapies. Furthermore, despite the notable synergistic efficacy of G&P combination therapy, in-depth investigations into the underlying immune synergy via cytokine profiling, antigen presentation detection, or transcriptome analysis will facilitate refinement of its mechanistic understanding. Finally, to fully highlight the translational potential of this promising strategy, future studies should focus on its long-term safety and the ability of this regimen to induce durable anti-tumor immune memory, which are critical for its future translation into clinical practice.

## Data Availability

The original contributions presented in the study are included in the article/supplementary material. Further inquiries can be directed to the corresponding author.
